# Delirium screening in the emergency department: evaluation and intervention

**DOI:** 10.1186/s13584-024-00603-1

**Published:** 2024-04-02

**Authors:** Tehilah Meged-Book, Reut Frenkel, Anna Nikonov, Vladimir Zeldetz, Amit Kosto, Dan Schwarzfuchs, Tamar Freud, Yan Press

**Affiliations:** 1 Faculty of Health Sciences, Ben-Gurion University of the Negev, Beer- Sheva, Israel; 2 Department of Internal Medicine, Soroka Medical Center, P.O.B. 151, Beer-Sheva, 84101 Israel; 3 Department of Geriatrics, Soroka Medical Center, Beer-Sheva, Israel; 4 Department of Pharmacy, Soroka University Medical Center, Beer Sheva, Israel; 5 Department of Emergency Medicine, Soroka University Medical Center, Beer-Sheva, Israel; 6 Siaal Research Center for Family Medicine and Primary Care, Faculty of Health Sciences, Ben-Gurion University of the Negev, Beer- Sheva, Israel; 7 Unit for Community Geriatrics, Division of Health in the Community, Ben-Gurion University of the Negev, Beer-Sheva, Israel; 8 Center for Multidisciplinary Research in Aging, Ben-Gurion University of the Negev, Beer-Sheva, Israel

**Keywords:** Delirium, Older adults, Emergency department

## Abstract

**Background:**

Between 8–17% of older adults, and up to 40% of those arriving from nursing homes, present with delirium upon admission to the Emergency Department (ED). However, this condition often remains undiagnosed by ED medical staff. We investigated the prevalence of delirium among patients aged 65 and older admitted to the ED and assessed the impact of a prospective study aimed at increasing awareness.

**Methods:**

The study was structured into four phases: a "pre-intervention period" (T0); an "awareness period" (T1), during which information about delirium and its diagnosis was disseminated to ED staff; a "screening period" (T2), in which dedicated evaluators screened ED patients aged 65 and older; and a "post-intervention period" (T3), following the departure of the evaluators. Delirium screening was conducted using the Brief Confusion Assessment Method (bCAM) questionnaire.

**Results:**

During the T0 and T1 periods, the rate of delirium diagnosed by ED staff was below 1%. The evaluators identified a delirium rate of 14.9% among the screened older adults during the T2 period, whereas the rate among those assessed by ED staff was between 1.6% and 1.9%. Following the evaluators' departure in the T3 period, the rate of delirium diagnosis decreased to 0.89%.

**Conclusions:**

This study underscores that a significant majority of older adult delirium cases remain undetected by ED staff. Despite efforts to increase awareness, the rate of diagnosis did not significantly improve. While the presence of dedicated delirium evaluators slightly increased the diagnosis rate among patients assessed by ED staff, this rate reverted to pre-intervention levels after the evaluators left. These findings emphasize the necessity of implementing mandatory delirium screening during ED triage and throughout the patient’s stay.

## Background

Delirium often serves as the sole indicator of a severe underlying medical condition in patients. The overall incidence of delirium in older adults ranges from 29–64% [1–6]. The incidence of delirium is highest in patients – 50–80% – after surgery, intensive care, and in geriatric, and hospital hospice wards. Between 8–17% of older adults and up to 40% of those who come to EDs from nursing homes present with delirium [7].

Delirium typically necessitates hospitalization, and it is considered unsafe to discharge patients with delirium, even if their acute illness appears to be minor [8]. The average hospital stay for older adults with delirium is twice as long as that for those without [9]. For those who present with delirium, there is a higher mortality rate in the 30 days after hospitalization (4–7% for those without delirium compared to 17% for those with delirium [10], and a higher rate of re-hospitalization within 30 days (27% compared to 13%) [11]. Another study indicated that while re-hospitalization rates increased, recurrent ED visits for older adults screened for delirium decreased by about 50% [12]. Other research showed a 37% mortality rate after 6 months for patients with delirium compared with 14% for those without – a rate that is 72% higher even after standardization regarding age, background diseases, severity of disease, functional dependence, and nursing home residence [9]. There is consensus that failing to diagnose delirium significantly escalates the risk of morbidity and mortality [8, 13].

Among patients requiring hospitalization, 18–35% are diagnosed with delirium upon admission. Older adults are at a high risk of developing delirium at the time of hospitalization, especially if they have underlying cognitive impairment. Prompt diagnosis of delirium is crucial [5, 8, 14, 15]. Studies have demonstrated that failing to diagnose delirium increases the risk of morbidity and mortality [8]and leads to greater functional decline post-discharge [13]. There is also evidence that non-pharmacological treatment of delirium during hospitalization can alleviate symptoms sooner, enhance cognition [16] and reduce the length of hospital stay [17, 18]. Combined intervention strategies, such as implementing a Delirium Room and components of the Hospital Elder Life Program, may mitigate some of the adverse outcomes associated with delirium, including functional loss, longer hospital stays, and increased mortality [19–21]. However, delirium often goes undetected by medical staff, with a diagnosis rate of only 15% of cases. This is attributed to the failure to screen for delirium and to document it as a diagnosis even when identified [6, 13, 22].

The diagnosis of delirium in patients aged 65 and older at Soroka University Medical Center [Soroka] a 1,200-bed tertiary medical center in southern Israel was evaluated in two retrospective studies reported in 2009 and 2015, respectively. In the first study, the screening rate was 12.5% with a 0% diagnosis rate [23]; in the second, following the addition of a geriatrician to the ED, the evaluation rate increased to 60.6% and the diagnosis rate to 3.8% [24]. However, a geriatrician has not been employed in this capacity in the past decade.

Previous studies focusing on increasing delirium screening and detection rates have shown that introducing the 4AT rapid clinical test for delirium increased the diagnosis rate. Further, after implementing education, auditing, and feedback, the rate of delirium screening improved significantly [23, 25]. Other studies have demonstrated the effectiveness of mandatory screening using an electronic medical record alert and follow-up reports [12].

The current study aimed to assess the rate of delirium diagnosis in the ED of a large teaching hospital, identify risk factors and predictors for delirium, and examine the impact of increasing awareness and the introduction of dedicated delirium evaluators on the diagnosis rate. This effort was made to develop a strategy and implement policy changes concerning delirium in the emergency department. Evaluating risk factors and predictors for delirium also aimed to aid in creating a focused screening tool for the relevant population.

## Methods

### Setting and subjects

**Fig. 1:**
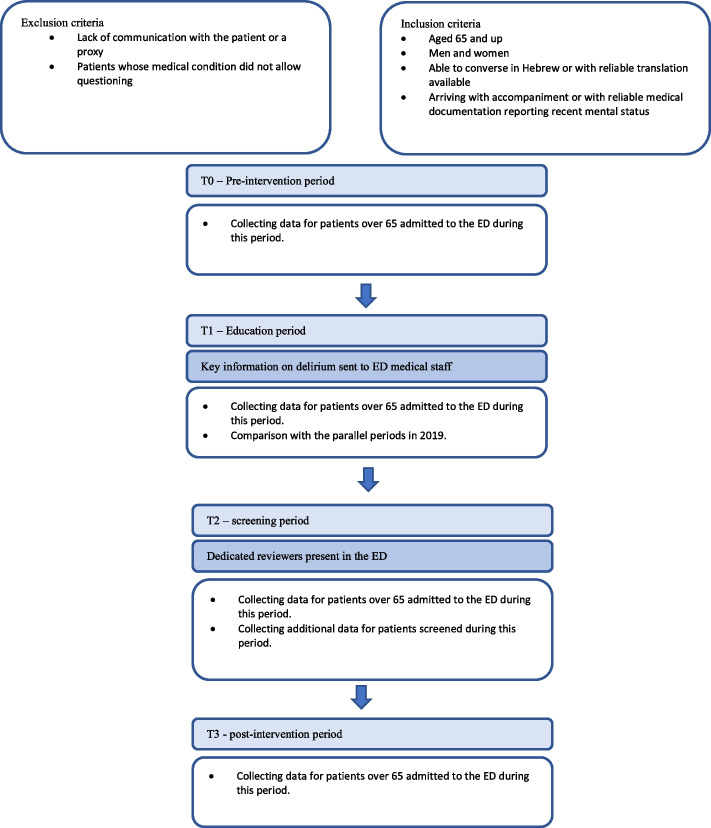
Study design

Soroka Hospital caters to a culturally diverse population, including Bedouins, other Arabs, and Jews from various sectors such as the general population, ultra-Orthodox, and Ethiopian communities. The main ED at Soroka is among the largest in Israel and the Middle East, offering services to everyone. It encompasses surgical and internal medicine departments, excluding obstetric and pediatric departments, which have their own separate EDs. On average, 470 patients are admitted daily, with 80 (approximately 18%) aged 65 and over (refer to Fig. 1 for Study Design).

1. The "pre-intervention period" (T0) spanned June-December 2019 and June-July 2021. Data were collected from Soroka's digital records to establish a baseline delirium rate. The parallel period in 2020 was excluded due to the exceptional circumstances of the COVID-19 pandemic. This comparison ensured no significant differences in ED routines between June-November 2019 and June-July 2021 that could affect the diagnosis rate.
2. The "awareness period" (T1), from August to October 2021, involved personal distribution and verbal explanation of information on delirium and its recognition to ED medical staff. A reminder featuring the Brief Confusion Assessment Method (bCAM) was posted in a highly visible area of the ED physician workstation for quick reference. Additionally, reminders about delirium screening and diagnosis were periodically sent to ED medical staff.
3. The "screening period" (T2), from November to December 2021, entailed screening of patients aged 65 and older admitted to the Soroka ED by a dedicated delirium evaluator. This evaluator informed the treating physician of positive delirium scores and updated the medical record and diagnosis list. The evaluator's role was limited to screening and did not extend to other diagnostic processes or medical decision-making. It is noted that due to the study's limitations, including evaluator availability, not all relevant patients were screened.
4. The "post-intervention period" (T3), from January 1–31, 2022, followed the T2 intervention period, during which dedicated evaluators no longer conducted screenings and were not present in the ED.

### Delirium screening method

Delirium, an acute and fluctuating cognitive state change characterized by inattention, disorganized thinking, and/or altered consciousness, is a clinical diagnosis assessed through specific tools, such as the bCAM [26]. Other tools include the 4 A's Test (4AT) [27], and the modified Confusion Assesment Method for the Emergency Department [mCAM-ED] [28]. The bCAM, already integrated into Soroka Medical Center's computerized system and familiar to medical staff, was selected for this study.

Inclusion criteria for the study were patients aged 65 or over capable of communicating in Hebrew, Russian, or Arabic. Excluded were those unable to communicate or in critical condition.

Two physicians, trained and supervised by an experienced geriatrician, conducted the screenings as delirium evaluators. They obtained verbal consent from patients or their proxies before administering the bCAM questionnaire, relying on the patient's proxy or medical record to ascertain if the confusion onset was acute. This consent process was approved by Soroka's Helsinki Committee (SOR-0487–20). Written informed consent for participation and publication was not required per legal and institutional guidelines.

### Sample size

The sample size calculation was based on the hypothesis that the delirium rate among patients presenting at the Soroka ED would be between 5–15%. Anticipating ED admission of approximately 2,000 patients aged 65 and over during the intervention periods and aiming for 80% power and a 95% confidence interval, at least 196 participants needed to undergo b-CAM screening. To compare patients with and without delirium across 30 variables in the bCAM assessment, we estimated 10–20 patients per variable, leading to the recruitment of 450 participants.

### Data collection

Data were collected from Soroka's digital system for all patients aged 65 and older across all study periods (T0-T3) and the corresponding periods in 2019. This included age, sex, delirium diagnosis (or equivalent terms like acute confusion, acute confusional state or confusion), number of hospitalizations, ED visits, and mortality within 90 days of the initial ED visit.

For patients evaluated with the bCAM questionnaire during the T2 period, additional data were collected, including marital status, type of residence, year of immigration, education and employment information, ED diagnosis, Charlson Comorbidity Score, vital signs upon ED admission, laboratory test results, medication list, alcohol use, drug use, smoking status, and ED discharge status.

### Statistical analysis

Statistical analysis was performed using IBM® SPSS® software, version 26 (SPSS, Inc., Chicago, IL). A *p*-value of < 0.05 was considered statistically significant. Patient demographic and clinical characteristics were compared between those screened positive and negative for delirium. The rate of delirium was compared across the retrospective (T0) and prospective (T1, T2, T3) study periods. Categorical variables were presented as frequencies and percentages. Continuous variables, such as age, were reported as mean ± standard deviation. A univariate analysis was performed to identify specific risk factors associated with delirium. The differences in categorical variables were assessed using the Fisher's exact test. Continuous data were compared using either the Mann–Whitney U test or the Student's t-test, based on whether the data distribution was non-normal or normal, respectively.

## Results

### Delirium diagnosis in ED: comparison of study periods

During the T0 period (June-December 2019 and June-July 2021), the ED staff identified an average of 15 cases of delirium per month out of 23,724 patients aged 65 and older admitted to the ED, representing less than 1% of admissions. There was no noticeable change in the rate of delirium diagnosis following the awareness intervention (T1). During the T2 period, 5,394 patients aged 65 and older were admitted to the ED, 4,943 of whom were generally assessed by ED physicians, not dedicated evaluators. The overall delirium rate among these patients increased to 1.6%, and the rate of delirium among patients independently assessed by ED physicians—when evaluators were present in the ED but did not actively participate in the screening process—was 1.9%. In the month following the study's conclusion (T3), the delirium rate reverted to less than 1% (Table 1).

**Table 1:** Delirium diagnoses in ED: comparison of study periods

Period	N of ED patients	N of delirium diagnosed	% delirium	*p* all	P T0/T1/T3
T0	23724	141	0.6	< 0.000001	0.082
T1	7451	49	0.7		
T2	4943	80	1.6		
T3	2471	24	0.89		

### Delirium diagnosis in the ED during the screening (T2) period: comparison of screened and unscreened patients

During the T2 screening period, 5,394 patients aged 65 and older were admitted to the ED. Of these, 1,231 were admitted when an evaluator was on duty, and 451 (37%) of these admissions were approached, consented, and screened for delirium using the bCAM. Unfortunately, the reasons for not screening some patients were not documented. Reasons included patients not being approached, some declining consent, or being unable to communicate. The characteristics of the three groups mentioned were similar (Table 2). The findings revealed that 67 (14.9%) out of the 451 patients screened by an evaluator tested positive for delirium.

**Table 2:** Delirium diagnosis in the ED during screening (T2) period: Comparison of screened and unscreened patients

	Screened for delirium by delirium evaluator	%	Unscreened (admitted at the time a dedicated evaluator was present in the ED)	%	*p* (451/780)	Unscreened (admitted during T2 period at the time a dedicated evaluator was not present in the ED)	%	*p* (451/4943)
*N*	451		780			4943		
Gender (female)	239	53	391	50.1	0.363	2566	51.9	0.696
Age, Mean ± SD	77.6 ± 8.1		77.3 ± 7.9		0.395	77.5 ± 8.0		0.644
Hospitalization	265	58.8	392	50.3	0.0047	2518	50.9	0.0017
Delirium Dx	67	14.9	15	1.9	< 0.00001	80	1.6	< 0.000001

Of the 451 patients screened by delirium evaluators, 296 (65.6%) were screened from 8 am to 4 pm, 124 (49.4%) from 4pm to 12 am, and 31 (6.9%) from 12 to 8 am. There were no significant differences in the delirium rate during these time-ranges.

### Comparison of delirious and non-delirious patients

As described in Table 3 patients diagnosed with delirium during the T2 period were older, more often unmarried, and had more comorbidities as indicated by a higher Charlson comorbidity score. The mean age for non-delirious patients was 77.3 ± 8.1, compared to 79.7 ± 8.1 for delirious patients (*p* = 0.0189). Of the 244 married patients, 99 (40.6%) were diagnosed with delirium, in contrast to 38 (59.4%) of the 172 unmarried patients (*p* = 0.0025). The mean Charlson comorbidity score was 3.0 ± 1.8 for patients with delirium, and 2.5 ± 2.0 for those without (*p* = 0.0337). Dementia was the only background disease significantly associated with delirium. Out of 68 patients diagnosed with dementia, 30 (44.1%) experienced delirium, compared to 37 (9.9%) without dementia (*p* < 0.000001).

**Table 3:** Patient characteristics – 451 patients screened

	**Delirium**		**No delirium**		***p***
	**67**		**384**		
	N	%	N	%	
**Shift**					
8:00–16:00	43	64.2	253	65.9	0.575
16:00–00:00	21	31.3	103	26.8	
00:00–8:00	3	4.5	28	7.3	
**Gender (female)**	37	55.2	202	52.6	0.794
**Age**					
Mean ± SD	79.7 ± 8.1		77.3 ± 8.1		0.0189
Range	65–105		65–97		
**Morbidity**	17	25.4	40	10.4	0.0029
**Hospitalization**	57	85.1	209	54.4	0.000002
**Confusion described by ED staff**	35	52.2	12	3.1	< 0.000001
**Family status**					
Married	26	40.6	218	61.9	0.0025
other	38	59.4	134	38.1	
**Living status**					
Alone	5	8.1	46	14.0	< 0.00001
Nursing 24/t at home	11	17.7	12	3.6	
LTC	25	40.3	12	3.6	
With family	21	33.9	259	78.7	
**Education**					
None/elementary school	14	38.9	47	19.8	0.057
High school	9	25.0	89	37.6	
Higher education	13	36.1	65	27.4	
**Vital signs**					
Temperature < 36.0 or > 37.5	21	32.8	23	6.7	0.0000001
Sys BP					
< 90	3	4.5	3	0.8	0.0055
90–140	33	49.3	141	37.0	
> 140	31	46.3	237	62.2	
Heart rate					
60–99	43	64.2	287	75.3	0.083
< 60 or > 99	24	35.8	94	24.7	
VAS 4 or more	5	10.2	88	25.1	0.0239
SAT < 93% or O2 support	15	22.4	42	11.1	0.0245
**Lab results**					
Hb (mg/dL)	12.6 ± 1.8		12.6 ± 2.3		0.61
WBC (× 10^3^/µL)	10.8 ± 5.6		9.0 ± 4.4		0.002
Neut (× 10^3^/µL)	8.5 ± 5.4		6.6 ± 4.0		0.0011
Lymph (× 10^3^/µL)	1.3 ± 1.0		1.6 ± 1.3		0.0199
Glucose (mg/dL)	178 ± 134.9		144.5 ± 65.1		0.0162
Urea (mg/dL)	64.5 ± 33.7		55.6 ± 35.8		0.0055
Creatinine (mg/dL)	1.5 ± 1.7		1.3 ± 1.26		0.0635
Sodium (mEq/L)	137.3 ± 6.9		137.8 ± 3.8		0.376
Potassium (mEq/L)	4.3 ± 0.7		4.4 ± 0.6		0.326
Calcium (mg/dL)	9.2 ± 0.7		9.3 ± 0.8		0.157
AST (U/L)	33.2 ± 26.8		38.1 ± 86.3		0.462
ALT (U/L)	21.8 ± 18.5		28.7 ± 81.7		0.731
GGT (U/L)	45.1 ± 88.3		57.3 ± 115.5		0.291
CRP (mg/L)	7.0 ± 7.9		3.5 ± 5.8		< 0.0001
**Comorbidities**					
Alcohol	1	1.5	3	0.8	0.953
Smoking	11	16.4	64	16.7	0.999
CHF	20	29.9	284	25.9	0.585
CVA	17	25.4	77	20.1	0.41
DM	36	53.7	166	43.3	0.149
Dementia	30	44.8	38	9.9	< 0.000001
**Charlson comorbidity score**	3.0 ± 1.8		2.5 ± 2.0		0.0337

Patients with delirium tended towards either hypothermia (body temperature lower than 36.0 °C) or hyperthermia (higher than 37.5 °C) – 32.8% with delirium versus 23% without (*p* < 0.0001), systolic blood pressure lower than 90 mmHg (4.5% vs 0.8%, *p* = 0.0055), and oxygen saturation lower than 93% or a need for supplemental oxygen (22.4% vs 11.1%, *p* = 0.0245). Conversely, non-delirious patients were more likely to have a systolic blood pressure higher than 140 mmHg (46.3% vs 62.2%, *p* = 0.0055) and a visual analog scale (VAS) score higher than 4 (10.2% vs 25.1%). In delirious patients, laboratory tests tended toward neutrophilia (with delirium 8.5 ± 5.4 × 10^3^/dL, 6.6 ± 4.0 × 10^3^/dL without, *p* = 0.0011), lymphopenia (with delirium 1.3 ± 1.0 × 10^3^/dL, 1.6 ± 1.3 × 10^3^/dL, *p* = 0.0199), increased urea (with delirium 64.5 ± 33.7 mg/dL, 55.6 ± 35.8 mg/dL without, *p* = 0.0055) and increased CRP (with delirium 7.0 ± 7.9 mg/L, 3.5 ± 5.8 mg/L without, *p* < 0.0001).

The hospitalization rate in the pre-intervention period (T0) was 63–65% for delirious patients, and 49–54% for those without. During the awareness intervention (T1), the rate for delirious patients was 79.6% and during the screening period (T2), the rate was 85.1%. Similar to the diagnosis rates, the T3 hospitalization rate returned to the baseline of 58.3%.

Among 451 screened patients, the mortality rate during the T3 follow-up was 25.4% for 67 patients with ED-diagnosed delirium, compared to 10.4% for the 384 patients without (*p* = 0.0029). The rate of revisits to the ED (0.3 ± 0.80, 3 ± 1.0) and rehospitalization (0.2 ± 0.6, 0.2 ± 0.5) were not significantly different between delirious and non-delirious patients.

## Discussion

This study aimed to enhance understanding of the delirium rate among older adults presenting at the ED of a 1,200-bed tertiary medical center in southern Israel and to examine the impact of a dedicated evaluator on the diagnosis rate. Our findings reveal that the delirium diagnosis rate by delirium evaluators was 14.8% when an evaluator was on duty in the ED (T2), aligning closely with prior studies that reported an average delirium rate of 8–17%, and up to 40% in older adults admitted to EDs [1, 3–6, 29].

No significant change in delirium diagnosis was noted between the T0 baseline period and the T1 awareness intervention. During the T2 screening period, the overall delirium rate in the ED, when assessed by ED physicians without a delirium evaluator present, increased to 1.6%, and to 1.9% when an evaluator was present. In the T3 post-intervention period, the delirium diagnosis rate by ED physicians reverted to below 1%, suggesting missed diagnoses rather than an actual increase in delirium prevalence during the T2 screening period.

Calculating the ratio of baseline delirium diagnosis rate to the rate found in this study, we found that the baseline rate of unrecognized delirium at the Soroka ED was around 75%. Other studies have put the figure at around 50% [13]. A 2003 retrospective study conducted at the Soroka ED [13], showed no diagnoses of delirium in the medical records of 319 older adults screened, indicating only 12.5% of older adults underwent an adequate mental status assessment by ED doctors [23]. The addition of a geriatrician to the ED staff increased the rate of mental status assessments to 60.6%, and the delirium diagnosis rate to 3.8%, still significantly lower than most literature reports [24].

We hypothesized that enhancing ED staff awareness about delirium and providing a diagnostic tool would increase the diagnosis rate. We anticipated a lasting effect and a significant rise in the diagnosis rate in the T3 follow-up period after dedicated evaluators were present in the ED for two months consistently reminding staff about delirium. However, our findings suggest that raising awareness alone does not effectively contribute to delirium recognition and intervention. This contrasts with the results reported by Martin et al. (2022) [25], where the introduction of the 4AT rapid clinical test for delirium increased the diagnosis rate from zero to 16%, and further interventions, including education, auditing, and feedback, raised the delirium screening rate to 92% [23]. Given the fact that the diagnosis rate returned to below 1% after the evaluators left the ED, and given the prior studies conducted at Soroka, we conclude that raising awareness alone, to the extent it has any effect, does not have a long-lasting effect on delirium diagnosis. Assigning a permanent evaluator to the ED may be cost-prohibitive and impractical. Thus, mandatory screening by nurses or physicians during triage, supported by electronic medical record alerts and follow-up reports, may offer a more viable and effective approach, as evidenced in previous studies [12].

Patients diagnosed with delirium were generally older, more isolated, and had a higher prevalence of comorbidities, particularly dementia, as indicated by a higher Charlson comorbidity score. This aligns with other research identifying higher age, comorbidity, and dementia as factors associated with delirium [30–32].

Our study also found that hospitalization rates for older adults with delirium were significantly higher than for those without throughout all study periods. The hospitalization rate increased slightly during the awareness intervention (T1) and reached its peak during the screening period (T2), consistent with prior research indicating high admission rates (55.9%) for delirious patients [9].

The mortality rate during the follow-up period (T3) was 2.4 times higher among older adults with delirium compared to those without. These results align with previous studies that reported similarly high mortality rates in patients with delirium, particularly within the first 6 months [14].

Kakuma et al. (2003) demonstrated that persons whose delirium was undetected by ED staff had the highest mortality rate over 6 months. The mortality rate of delirious persons detected in the Soroka ED was much lower, and not significantly different from that of those without [8]. In our study, for ethical reasons, treating physicians were always informed when screening results for delirium were positive, and the diagnosis was documented in the medical record. Consequently, we cannot directly compare our results with those of Kakuma et al. However, Kakuma's study suggests that the act of diagnosing delirium itself can reduce mortality.

We recommend the implementation of mandatory screening in the ED during the triage of patients aged 65 and over. This initiative will be launched at Soroka Medical Center, where its effectiveness will be assessed and evaluated. The geriatric staff will provide consultations during this period. An essential part of this process will involve making the bCAM, already integrated into the hospital’s computerized system, a mandatory step that cannot be bypassed before finalizing a patient's chart.

Previous studies have shown that a small but significant proportion of adults initially not identified as delirious were found to be so upon retesting three hours later. This underscores the potential benefit of conducting delirium screenings at multiple points during a patient’s ED visit [33]. Given that the median stay in the Soroka ED is 2.7 h [34], screening will initially occur during triage, with notifications for re-screening appearing every three hours for patients whose stay exceeds this duration.

Performance will be assessed through electronic reports sent to the ED director and the principal investigator or program director, and these reports will be shared with the ED staff. We anticipate that this intervention will form part of a larger effort to improve the hospital's age-friendliness and that the enhanced detection of delirium will encourage the adoption of broader initiatives to improve geriatric patient care. Successful interventions elsewhere, such as a Delirium Room [19] and components of the Hospital Elder Life Program [21], could be adapted for the ED setting or after hospital admission to improve patient outcomes [19–21].

Our study's strengths include a relatively large sample size, enhancing the reliability of our findings regarding the ED delirium rate and its clinical correlations. We also report on an unsuccessful intervention, guiding us to explore more effective strategies. However, this study has limitations, including that only 36.6% of eligible patients were screened, with no documented rationale for exclusions, possibly omitting non-communicative patients. Second, we used Charlson's Comorbidity Index to assess comorbidity, but it does not factor in the severity for each disease included in the index, except for diabetes mellitus and liver disease, and thus may not be optimal in understanding risk factors for delirium. Most importantly, we did not follow patients after they were hospitalized or discharged, and this study did not include interventions after delirium was diagnosed. Therefore, we do not have data concerning if and how the patients’ delirium resolved, nor mortality data beyond 90 days after hospital admission.

## Conclusions

Delirium significantly increases the risk of morbidity and mortality, yet most cases in the ED remain undiagnosed, even after interventions designed to raise awareness and the temporary introduction of dedicated evaluators. We conclude that short-term educational interventions are largely ineffective. Therefore, implementing mandatory screening by nurses or physicians during triage, supported by electronic medical record alerts and follow-up reports, is crucial for the diagnosis and subsequent management of delirium. Detecting delirium allows for the application of validated interventions to improve patient outcomes.

## Data Availability

The datasets during and/or analyzed during the current study available from the corresponding author on reasonable request.

## Abbreviations

ED: Emergency department
ED staff: Emergency department medical staff
bCAM: Brief Confusion Assessment Method
4AT: 4 A’s Test
mCAM-ED: Modified Confusion Assessment Method for the Emergency Department
